# Nematic Order, Plasmonic Switching and Self‐Patterning of Colloidal Gold Bipyramids

**DOI:** 10.1002/advs.202102854

**Published:** 2021-09-20

**Authors:** Zhijian Mai, Ye Yuan, Jung‐Shen B. Tai, Bohdan Senyuk, Bing Liu, Hao Li, Yao Wang, Guofu Zhou, Ivan I. Smalyukh

**Affiliations:** ^1^ Guangdong Provincial Key Laboratory of Optical Information Materials and Technology National Center for International Research on Green Optoelectronics Institute of Electronic Paper Displays South China Academy of Advanced Optoelectronics South China Normal University Guangzhou 510006 P. R. China; ^2^ Department of Physics and Soft Materials Research Center University of Colorado Boulder CO 80309 USA; ^3^ Materials Science and Engineering Program Department of Electrical, Computer and Energy Engineering University of Colorado Boulder CO 80309 USA; ^4^ Renewable and Sustainable Energy Institute National Renewable Energy Laboratory and University of Colorado Boulder CO 80309 USA

**Keywords:** bipyramid, electric switching, gold nanoparticles, nematic order, surface plasmon resonance, topological soliton

## Abstract

Dispersing inorganic colloidal nanoparticles within nematic liquid crystals provides a versatile platform both for forming new soft matter phases and for predefining physical behavior through mesoscale molecular‐colloidal self‐organization. However, owing to formation of particle‐induced singular defects and complex elasticity‐mediated interactions, this approach has been implemented mainly just for colloidal nanorods and nanoplatelets, limiting its potential technological utility. Here, orientationally ordered nematic colloidal dispersions are reported of pentagonal gold bipyramids that exhibit narrow but controlled polarization‐dependent surface plasmon resonance spectra and facile electric switching. Bipyramids tend to orient with their C_5_ rotation symmetry axes along the nematic director, exhibiting spatially homogeneous density within aligned samples. Topological solitons, like heliknotons, allow for spatial reorganization of these nanoparticles according to elastic free energy density within their micrometer‐scale structures. With the nanoparticle orientations slaved to the nematic director and being switched by low voltages ≈1 V within a fraction of a second, these plasmonic composite materials are of interest for technological uses like color filters and plasmonic polarizers, as well as may lead to the development of unusual nematic phases, like pentatic liquid crystals.

## Graphical abstract

Orientationally ordered nematic colloidal dispersions of pentagonal gold bipyramids exhibit narrow but controlled polarization‐dependent surface plasmon resonance spectra and facile electric switching. Bipyramids tend to orient with their C_5_ rotation symmetry axes along the nematic director, exhibiting spatially homogeneous density within aligned samples. These plasmonic composite materials are of interest for technological uses like color filters and plasmonic polarizers.

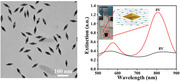

## 1 Introduction

Designing composite materials with pre‐engineered physical properties typically requires controlling structure and chemical composition of the constituents on the nanometer‐to‐micrometer scales.^[^ ^1^, ^2^, ^3^ ^]^ The ensuing physical behavior then can lead to the emergence of previously unrealized material functionality.^[^ ^1^ ^]^ An example of such an approach is a ferromagnetic colloidal material formed by mesoscale self‐assembly of magnetically monodomain anisotropic colloidal nanoparticles within a liquid crystal (LC) host medium that leads to the emergence of fluid ferromagnetic colloidal order.^[^ ^3^, ^4^, ^5^, ^6^, ^7^ ^]^ Furthermore, various ordered assemblies of nanoparticles in LCs may enable composites with properties that are not only uncommon for both the LC molecular and colloidal constituents, but that can be also tuned by fields, light, and other external stimuli.^[^ ^8^, ^9^, ^10^, ^11^, ^12^, ^13^, ^14^, ^15^, ^16^, ^17^, ^18^ ^]^ This may lead to technological applications ranging from smart windows to electro‐optic and photonic devices,^[^ ^12^, ^13^ ^]^ as well as to a fertile ground for new fundamental science.^[^ ^3^ ^]^ In the latter case, combining colloidal nanoparticles with nematic fluid hosts already led to the discovery of ferromagnetic,^[^ ^4^, ^5^, ^6^, ^7^ ^]^ orthorhombic,^[^ ^10^ ^]^ and monoclinic^[^ ^19^ ^]^ nematic LC order and triclinic and other colloidal crystals,^[^ ^9^ ^]^ but potentially even a much larger range of possibilities can be accessed by dispersions of nanoparticles with various symmetries and topological characteristics. However, realization of diverse mesoscale soft matter composites is hindered by a limited inventory of nanoparticles demonstrated to form stable LC colloidal dispersions with orientational correlations between the anisotropic nanoparticle and the host medium. For example, the long‐range ordered LC dispersions of anisotropic colloids, such as gold, silver, and other metal nanoparticles, were so far limited mainly to rods and platelets.^[^ ^12^, ^13^ ^]^

In this study, we describe nematic LC colloids formed by gold nanoparticles shaped as pentagonal bipyramids (GNPBs) with *D*_5h_ point group symmetry. When homogeneously dispersed in a uniaxial *D*_∞h_ nematic LC fluid host, GNPBs orient with their C_5_ rotation symmetry axes along the nonpolar nematic LC director, **N**≡‐**N**, describing the average orientation of the LC's constituent rodlike molecules. The ensuing composite materials exhibit polarization‐dependent surface plasmon resonance (SPR) spectra that can be controlled by facile electric switching. As compared to colloidal dispersions of plasmonic nanorods, consistent with the studies for isotropic fluid host media,^[^ ^20^, ^21^, ^22^, ^23^, ^24^ ^]^ GNPBs exhibit high monodispersity of geometric dimensions and provide more narrow surface resonance spectra. In the form of nematic colloidal dispersions, GNPBs exhibit electrically reconfigurable SPR spectra with electrically controlled spectral shifting of SPR peaks. We show how topological solitons, like heliknotons,^[^ ^25^ ^]^ allow for spatial reorganization of these nanoparticles according to the elastic free energy density landscape within them. Numerical modeling explains how elastic free energy landscape allows for tuning the nanoparticle density within the dispersions containing heliknotons, as well as captures the overall physical behavior of orientationally ordered plasmonic LC composites. Finally, we discuss how these plasmonic composite materials are of interest for uses in technological applications and in the fundamental exploration of coexistence of order and fluidity within the soft matter systems.

## 2 Results and Discussion

### 2.1 Orientated dispersions of GNPBs

**Figure 1:**
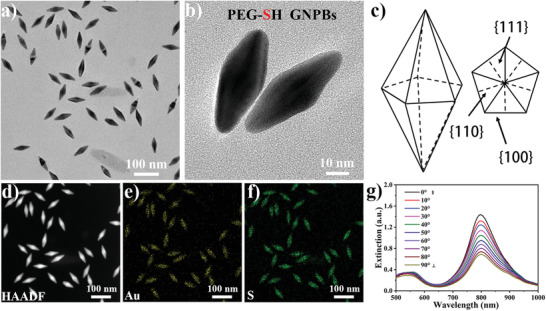
Structure, symmetry and surface plasmon resonance spectra of GNPBs. a,b) TEM images of PEG‐SH capped GNPBs. c) Schematic illustration of the crystallographic planes of a GNPB. The right side is the base cross‐section, showing the {111} twinning planes, {110} planes, and stepped {100} facets. d) High‐angle annular dark‐field (HAADF)‐STEM image. e,f) Energy dispersive spectrometer (EDS) images of PEG‐SH capped GNPBs show the elemental mapping of Au element (e) and S element (f) demonstrating the successful surface capping of GNPBs with PEG‐SH. g) Polarization‐dependent extinction spectra of shear‐aligned composite of the cellulose nanorods and GNPBs obtained for different polarizations of incident light with respect to the shearing direction.

Our pentagonal GNPBs, shown in **Figure** **1**, have C_5_ rotation symmetry axis and typical monodisperse dimensions of 35 × 70 nm in directions perpendicular and parallel to it, respectively. The crystallographic planes of the facetted GNPBs are marked in Figure 1c, which provides the perspective views on these particles along directions perpendicular and parallel to the C_5_ symmetry axis. Surface functionalization of GNPBs with PEG‐SH preserves geometry of these particles while also providing a uniform surface grafting of these polymer molecules on all their facets (Figure 1a–f). While SPR properties of GNPBs have been studied both experimentally and theoretically,^[^ ^20^, ^21^, ^22^, ^23^, ^24^ ^]^ these previous works revealed and explained how GNPBs exhibit polarization dependence of SPR at the level of individual nanoparticles but do not retain such polarization dependence properties within colloidal dispersions in isotropic solvents. Figure 1g shows how alignment and polarization dependence of SPR can be imposed by unidirectional shearing. In this case, GNPBs are aligned by shearing their codispersions with much longer cellulose nanorods to eventually form dried solid films with the alignment of both cellulose nanoparticles and GNPBs, using an approach described in detail elsewhere.^[^ ^26^ ^]^ While this and other approaches can be used to align GNPBs, similar to how this was done for gold nanorods and other anisotropic nanoparticles,^[^ ^26^ ^]^ such plasmonic nanocomposites lack switching in response to external fields, achieving which is one of the goals of this work.

**Figure 2:**
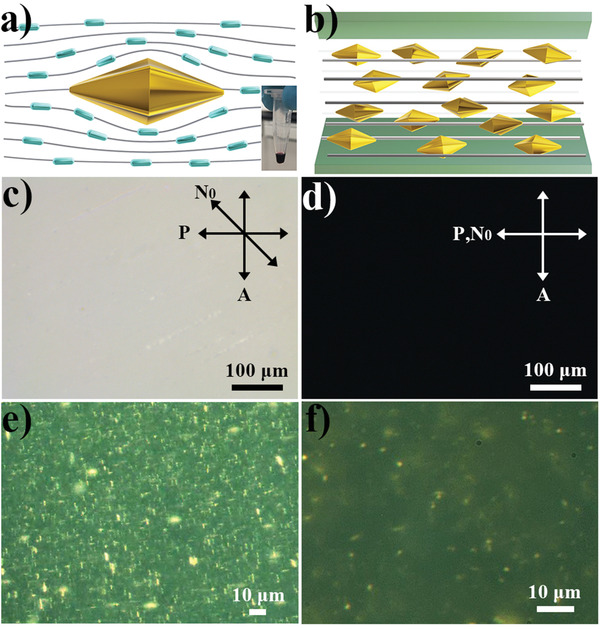
Oriented colloidal dispersions of GNPBs in a nematic LC. a) Schematic illustration showing a single GNPB oriented by the nematic host medium through a combination of surface anchoring interactions and minimization of **N**(**r**)‐distortions. b) Schematic of GNPBs in a LC following **N**_0_. c,d) POM images of the LC‐GNPB composite with **N**_0_ at 45° (c) and 0° (d) to the incident polarization **P**. e,f) Darkfield microscopy images showing the colloidal dispersion nature of the LC‐GNPB composite; **N**_0_ is oriented along the vertical edge of images.

**Figure 3:**
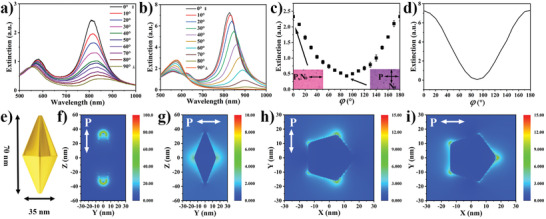
Characterization and modeling of polarization‐dependent surface plasmon resonance effects in LC‐GNPB composites. a,b) Experimental (a) and simulated (b) extinction spectra of a LC‐GNPB composite measured for different angles *φ* between the incident light's linear polarization and **N**_0_. c,d) Measured (c) and simulated (d) extinction of long‐wavelength SPR peaks versus *φ*. e) The size of the GNPB is chosen to be 35 nm by 70 nm and the radius of the rounded tips is taken to be 3.5 nm, consistent with the TEM images. f–i) Simulated plasmonic enhancement of light at 807 nm near a GNPB. f–g) Orientation of the light propagation direction is perpendicular to the GNPB's C_5_ axis and the incident polarization is parallel (f) and perpendicular (g) to the long axis of the GNPB. h–i) Orientation of the light propagation direction is parallel to the GNPB's C_5_ axis and the incident polarization is parallel (h) and perpendicular (i) to one of the sides of the pentagon where facetted surfaces meet. The colored scale bars represent the logarithm of the relative light intensity, i.e., log(*I*/*I*_0_), where *I* is the light intensity around the particle and *I*_0_ is the incident light intensity.

When dispersed in nematic LC hosts like pentylcyanobiphenyl (5CB), GNPBs align with their C_5_ rotation symmetry axes along the local nematic director field **N**(**r**) with the nonpolar head‐tail symmetry (**Figure** **2**a,b). This spontaneous alignment stems from the minimization of the overall free energy of the LC host medium in presence of such nanoparticles, including the surface anchoring energy due to finite/weak tangential surface boundary conditions on the colloidal facets and weak elastic distortions of **N**(**r**) around the particles (Figure 2a). At used concentrations below 1% by volume, such spontaneous alignment of C_5_ axes with the nematic director is followed by all individually dispersed nanoparticles and is set to be monodomain unidirectional along the LC's far‐field director **N**_0_ defined by using the surface treatment (Figure 2b). This behavior of monodomain LC‐GNPB orientational ordering within the colloidal dispersion is confirmed with polarizing optical microscopy (Figure 2c,d) while rotating **N**_0_ between two crossed polarizers of the microscope. Dark field images taken at different concentrations of GNPBs (Figure 2e,f) reveal that the nanoparticles are (mostly) individually dispersed, with occasional small aggregates caused by dust inclusions and confining surface imperfections. GNPBs exhibit no positional correlations of centers of mass within the dispersion, as revealed by the centers of scattering from these sub‐diffraction‐limited objects in the dark field optical imaging mode (Figure 2e,f). This, combined with orientational ordering (Figures 2 and 3) of C_5_ axes along **N**(**r**), shows that the “induced” orientational ordering of GNPBs mimics that of the nematic host and has *D*_∞h_ point group symmetry despite of the lower symmetry of GNPBs.

Inch‐scale unidirectional self‐alignment of GNPBs in the nematic LC along the far field director **N**_0_ results in polarization‐dependent SPR properties of the entire LC‐GNPB samples (**Figure** **3**). SPR spectra change dramatically with rotating the polarization of incident light relative to **N**_0_ by 90° (Figure 3a,b). Furthermore, this evolution of polarized SPR spectra of the LC‐GNPB composite is markedly different from that obtained solely via the shear‐induced unidirectional alignment of GNPBs on a surface (Figure 1g). Unlike in the thin coatings of orientationally ordered GNPBs (Figure 1g), where the effects of birefringence due to medium around the nanoparticles can be neglected, here the SPR spectra feature not only much stronger changes of intensities of different SPR modes, but also significant shifts of the SPR peaks (Figure 3a,b). Interestingly, the extinction peak of the short‐wavelength SPR mode blue‐shifts whereas that of the long‐wavelength mode red‐shifts (Figure 3a), which is related to the change of an effective refractive index seen by light while traversing the LC‐GNPB composite. The extinction peaks exhibit angular variations (Figure 3c) with the angle *φ* between the linear polarization of incident light **P** and the direction of ordering **N**_0_ of both the LC host and GNPBs, consistent with the extinction being roughly proportional to (**P**•**N**_0_)^2^. This extinction variation is accompanied with the change of coloring of the LC‐GNPB composite observed in transmission (insets of Figure 3c; Video S1, Supporting Information). Such behavior can be closely reproduced in numerical modeling of the SPR spectra of GNPBs within a surrounding medium described by an optical‐frequency dielectric tensor with material constants corresponding to those of the used 5CB (Figure 3b,d). The slight differences seen when comparing experimental (Figure 3a) and computer‐simulated SPR spectra versus the angle *φ* can be attributed to the fact that the scalar orientational order parameter of GNPB in the experiments is finite, even though estimated to be relatively high [*S* = (*A*_||_−*A*_⊥_)/(*A*_||_+2*A*_⊥_) = 0.68, where *A*_||_ and *A*_⊥_ are the long‐wavelength extinction peaks when **P** is, respectively, at 0° and 90° with respect to **N**_0_], whereas our modeling, for simplicity, assumes perfect orientational order when this parameter would be unity and is done for a single GNPB for just one orientation of the pentagonal base relative to the light's incidence direction and polarization. The other source of small discrepancies originates from the fact that the nematic host medium alters the polarization state of light traversing through the sample when **P** of the incident light is at orientations in‐between 0° and 90° relative to **N**_0_. Computer‐simulated for the geometry shown in Figure 3e, the SPR enhancement patterns (Figure 3f–i) highlight the hot spots of the strongest plasmonic enhancement at the tips and other sharp geometric features of the GNPBs. This indicates that the experimental SPR spectra with both red (for the long‐wavelength peak) and apparent blue (for the short‐wavelength peak) shifts of the SPR peaks originate from both the polarization dependence of an effective refractive index of the LC host as well as from the geometry of bipyramids relative to the LC's far‐field director and light propagation direction. Consistent with this qualitative explanation, numerical modeling fully reproduces the experimentally observed red sifting (Figure 3). Modeling of the short‐wavelength band for a single orientation of a bipyramid reveals a more complex spectral features, which slightly differ from the experimental counterpart because experimental dispersions have multiple GNPBs at different azimuthal orientations, as well as with the finite scalar order parameter describing their orientational ordering, thus causing an effective averaging of some of these spectral features.

### 2.2 Electric switching

**Figure 4:**
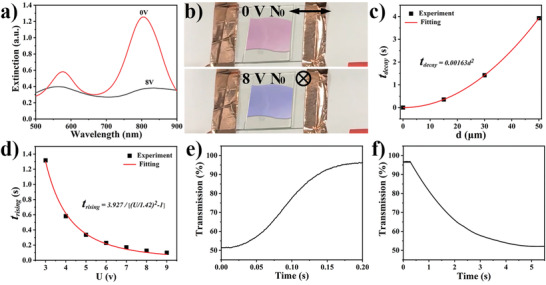
Electric switching of the LC‐GNPB colloidal dispersions. a) Extinction spectra for **P** along the rubbing direction with and without external electric field application. b) The corresponding color change during switching is also visible for unpolarized incident light. c) Decay time versus thickness of the cell *d*. d) Rising time versus an applied voltage *U*. Fitting expressions are shown next to the fitting curves in (c,d). e,f) Transmission versus time upon the application (e) and removal (f) of the external field (*U* = 8 V) applied to the LC‐GNBP cell with *d* = 50 µm.

SPR spectra of the LC‐GNPB composites can be switched by applying low voltages in the range 1–10 V at frequencies ≈1 kHz (**Figure** **4**a,b), where both short‐ and long‐wavelength plasmonic modes exhibit significant variations with applied voltage (Figure 4a). Similar to the response of pristine LCs, the LC‐GNPB composite exhibits the rising time decreasing with the amplitude of the applied voltage *U* and the decay time scaling quadratically with the sample thickness (Figure 4c–f). The threshold voltage of the composite cell extracted from fitting the dependency of rising time versus *U* is ≈1.42 V, while the typical rising and decay times for a 50‐µm‐thick cell are 0.127 and 3.927 s at 8 V, respectively, as shown in Figure 4. The numbers are comparable to the ones estimated for pristine 5CB.^[^ ^12^ ^]^ Such behavior is natural as the orientations of the GNPB axes are coupled to the **N**(**r**) in the process of switching, similar to what was previously demonstrated for gold nanorods.^[^ ^12^, ^13^ ^]^ The electrically controlled change of color of the composite reflects the fact that the long‐wavelength SPR peak is primarily in the near infrared range (Figure 4b; Video S2, Supporting Information), so that the color changes relate mainly to the variations of the short‐wavelength SPR peak of the spectra.

Dispersion of nanoparticles can change the viscoelastic properties of a LC‐GNPB composite comparing to the pristine host medium like 5CB. Rotational viscosity *γ*_1_ ≈ 103 mPa⋅s of our LC‐GNPB composite determined from fitting the experimental relaxation data (Figure 4c) with an expression *t*_decay_ = (*γ*_1_/*K*_11_)(*d* ^2^/*π* ^2^), where *K*_11_ = 6.4 pN is a splay elastic constant of 5CB, is somewhat increased comparing to *γ*_1_ ≈ 81 mPa s of a pristine 5CB due to a concentrated dispersion of GNPBs. At the same time, elastic constants of the LC‐GNPB composite can also change by ≈10% comparing to a pristine 5CB due to interactions between the individual nanoparticles with the embedding solvent through surface‐anchoring forces and colloidal interactions between nanoparticles themselves.^[^ ^27^ ^]^ Overall, our findings reveal that doping of the LC with GNPBs only modestly changes the LC‐GNPB composite's switching characteristics as compared to that of the LC host medium.

### 2.3 Spatial patterning with heliknotons

**Figure 5:**
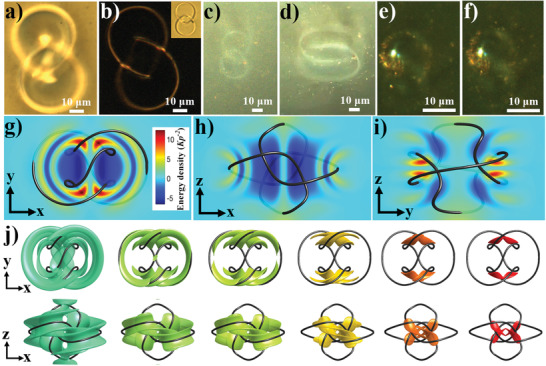
Spatial self‐patterning of nanoparticles within topological solitons. a) Brightfield and b) polarizing optical images of a heliknoton in a cholesteric LC at *U* = 6.16 V. In (b), crossed polarizers are oriented along the image edges, with the inset showing an image between parallel polarizers along the vertical image edges. c–f) Dark‐field optical images of a heliknoton showing the templated spatial positions of GNPBs taken at different voltages of 6.16V (c) and 8.16V (d) and 6.16V (e,f), with the last two images of the same heliknoton taken at different depths of the sample, about 10 micrometers apart. Note how the spatial trace of patterned GNPBs is morphing in response to changing the applied voltage (c,d), along with the electric reconfiguration of the heliknoton and the energetically costly region within its structure. The sample thickness *d* and cholesteric pitch *p* are, respectively, 100 µm and 40 µm in (a–d) and 50 µm and 20 µm in (e,f). g–j) Energetics (g–i) and topology (j) of the localized structure of a heliknoton. 3D energetic profile of a chiral LC‐GNPB heliknoton is visualized by colors corresponding to free energy density values shown (g–i) in orthogonal cross‐sections and (j) by isosurfaces corresponding to energy densities of 2, 3.5, 4, 6, 8, 10 (left to right; in units of *Kp* ^−2^, where *K* is an average elastic constant of 5CB). The color scale is shown in the inset in (g). The singular vortex line in a helical axis field ****χ**** (***r***) closing into a trefoil knot is depicted as a black tube.

The above examples demonstrate spatially‐invariant self‐ordering of GNPBs directed by the nematic host and LC‐mediated switching of nanoparticle orientations. However, various future applications of mesostructured LC‐GNPB composites may also require spatial modulation of density and orientation of the nanoparticles along with a facile response to external stimuli. An approach involving singular topological defects, like disclinations and point defects, can be used to accomplish this goal when localization within small spatial regions of ≈10 nm is needed, along line‐like or within point‐like singularities.^[^ ^28^, ^29^, ^30^, ^31^, ^32^ ^]^ **Figure** **5** shows how topological solitons called “heliknotons,”^[^ ^25^ ^]^ elements of the 3^rd^ homotopy group *π*_3_(S ^2^/ℤ_2_) = ℤ [for a vectorized director field, *π*_3_($S^{2}$) = ℤ], with Hopf index being a relevant topological invariant, can be used to control nanoparticle density within reconfigurable spatial regions on nanometers‐to‐micrometer scales. A series of transmission‐mode polarizing and dark‐field optical images show that the local density of nanoparticles can be locally increased within some localized regions of the heliknotons (Figure 5a–f). Unlike in the case of singular topological defects, within which **N**(**r**) cannot be defined within small nanoscale regions, **N**(**r**) is continuous within the entire volume of these topological solitons. Consequently, there is no segregation of the nanoparticles into singular defect regions and thermal energy drives their diffusion within the volume of heliknotons. However, the GNPBs are found interacting with the elastic energetic landscape as the density of dispersed nanoparticles is found to be higher in certain regions within heliknotons corresponding to the largest values of free energy density (Figure 5). The overall LC medium's elastic free energy can be reduced when weak perturbations of **N**(**r**) around the GNPBs overlap with certain types of elastic **N**(**r**)‐distortions within the larger‐scale pattern of heliknoton (Figure 5). These elastic energy gradients drive self‐patterning of GNPBs within these topological solitons, effectively making golden‐GNPB‐decorated solitonic knots (Figure 5). The calculated elastic free energy landscapes of heliknotons are consistent with the denser regions of GNPB nanoparticles within their volume (compare images and free energy density plots in Figure 5). This shows how heliknoton's interaction with nanoparticles differs from that of singular defects, where the most energetically costly regions are associated with the defect's singular cores and, typically, one observes energy density continuously decreasing with the distance from the singular core. Also differently from singular defects, the probability of finding GNPBs within different regions of the heliknoton depends on applied voltage, which potentially could be used to dynamically reconfigure this behavior.

## 3 Discussion and Conclusions

An interesting question emerging from our findings is what are the fundamental consequences of orienting the *C*_5_ rotation symmetry axes of colloidal GNPBs with **N**(**r**) within nematic colloidal dispersions? One can imagine that, under suitable conditions, the additional in‐plane orientational ordering could emerge in the plane orthogonal to **N**_0_, potentially giving origins to pentatic LCs with uninhibited fluidity. Nematic colloidal quasicrystals could emerge as yet another possibility. Colloidal particles with shapes of pentagonal prisms and truncated pyramids have attracted considerable recent interest because of the possibility to self‐assemble nematic colloidal quasicrystals and other configurations that go beyond crystalline order of colloids.^[^ ^33^, ^34^, ^35^ ^]^ However, experimental realization of such colloidal quasicrystals within nematic fluid hosts has been challenged by a strong, thermally‐irreversible elastic binding due to the facts that the colloidal particles had micrometer‐range dimensions and strong surface boundary conditions.^[^ ^33^, ^34^, ^35^ ^]^ The advantage of our nematic GNPB colloids is that the particles with nanoscale dimensions induce weaker elastic distortions and, thus, colloidal assemblies can thermalize in ground‐state configurations. Thus, the demonstrated stable colloidal dispersion of GNPBs in nematic hosts could potentially lead to experimental realization of exotic condensed matter states that combine fluidity with various types of long‐range orientational order and possibly also with quasicrystal‐like organization.

From the technological applications standpoint, because of the sharp features and ensuing plasmonic hot spots (Figure 3f–i), LC‐GNPB composites are particularly attractive as reconfigurable SPR‐enhancing materials. One can envisage using this plasmonic enhancement to tune luminescence intensity and lifetime of dye molecules or quantum dots co‐dispersed within the nematic host, as well as design a host of other effects based on plasmon–exciton interactions.^[^ ^36^ ^]^ By varying geometric features of GNPBs, the SPR peaks can be tuned across the visible and near infrared parts of the electromagnetic spectrum, so that electric switching of such spectra could be used in designing smart and privacy windows with controlled visible transparency and near‐infrared‐range solar gain control.^[^ ^12^, ^13^ ^]^ Increasing the inventory of plasmonic nematic colloids is also of interest for various designs of reconfigurable optical metamaterials.^[^ ^37^ ^]^

In summary, we have developed a colloidal system comprising pentagonal gold bipyramids and a nematic LC host medium. This organic–inorganic composite is exhibiting an emergent effective‐medium behavior like orientational mimicry of nonpolar molecular nematic order by the nanoparticles and switchable, polarization‐sensitive surface plasmon resonance properties of the composite. Using topological solitons dubbed “heliknotons,” we have demonstrated the feasibility of diffuse spatial self‐patterning of nanoparticle positions, where the local density of GNPBs can be modulated by exploiting the elastic free energy landscape sensed by the nanoparticles. We envisage a host of fundamental science and technological uses of our colloidal bipyramid dispersions, ranging from 3D optical metamaterials and switchable privacy windows to realization of unusual low‐symmetry LCs and quasicrystals.

## 4 Experimental Section

### Synthesis and Surface Functionalization of Nanoparticles

GNPBs were prepared by a two‐step synthesis process, including preparation of gold seeds and growth of gold bipyramids. The first stage involved the following steps. The mixture of 4 mL of HAuCl_4_ (0.5 × 10^−3^ m), 4 mL of cetyltrimethylammonium chloride solution (CTAC, 95 × 10^−3^ m), and 72 µL of HNO_3_ (250 × 10^−3^ m) were added into a 20 mL vial at room temperature. Then, 100 µL of fresh NaBH_4_/NaOH (1/1, 50 × 10^−3^ m) solution was added to the mixture quickly under vigorous stirring (at about 800 rpm). During this process, the color of the mixture gradually changed from yellow to brown. It was then continued to stir for one minute. After adding 16 µL of citric acid (1 m) to the mixture, the vial was closed and placed into the oil bath (80–85^ ^°C) for 1 h until the color of the mixture changed to fuchsia, indicating the generation of the gold seeds. It was then proceeded with the second stage of the synthesis, involving growth of gold bipyramids. Aqueous solutions of 160 µL of HAuCl_4_ (25 × 10^−3^ m) and 16 mL of cetyltrimethylammonium bromide (CTAB, 47 × 10^−3^ m) were mixed in a 20 mL vial, yielding a deep‐yellow‐colored solution. While it was stirred at 800 rpm, 72 µL of AgNO_3_ (10 × 10^−3^ m) solution was added, followed by further addition of 160 µL of 8‐hydroxyquinoline (0.4 m, in ethanol) solution. The deep‐yellow color of the gold solution disappeared, turning colorless immediately. Then, 160 µL of gold seeds solution (obtained in the previous step of synthesis) was added into the growth solution. After 1 min of stirring, the vial was put into a water bath (40–45^ ^°C). After 15 min, 100 µL of 8‐hydroxyquinoline (0.4 m) was added into the mixture and let staying for another 60 min at 40–45^ ^°C. The color of the obtained gold bipyramids solution turned to deep purple. Finally, the obtained GNPBs dispersion was centrifuged at 10 000 rpm for 10 min to eliminate the excessive surfactant and washed by deionized water twice.

To surface‐functionalize the synthesized GNPBs, the following was done. First, 1 m NaOH solution was used to adjust the pH of the gold bipyramids solution to become 12. Then, the dichloromethane (GNPBs dispersion/dichloromethane—1:1v/v) was introduced into the solution, followed by adding 5 kDa mPEG‐SH (1 mg mPEG‐SH per 1 mL GNPBs dispersion with optical density of 4). The two‐phase system formed, with vertical separation caused by the density difference between dichloromethane and water. During standing for at least 24 h, CTAB ligands on the bipyramids surface were replaced by mPEG‐SH, which binds to the GNPBs surface through a strong Au–S covalent linkage. Ligand exchange can be visually observed as a change in color of the aqueous and organic phases, indicating that the GNPBs were phase‐transferred from aqueous to organic phase. The organic phase was separated and washed by methanol via centrifuging at 10 000 ppm for 10 min three times to obtain a purple PEG‐coated GNPBs dispersion. The shape, size and composition of the ensuing GNPBs was confirmed by analyzing the images obtained from transmission electron microscopy (TEM).

### Sample Preparation

The colloidal dispersions of GNPBs in LCs were prepared by mixing 100 µL of GNPBs in methanol with 30 µL of LC and evaporating the solvent in an oven at 75 °C for 1 h. The resultant isotropic mixture was sonicated in a water bath at 75 °C for 5 min and then quenched to nematic phase while agitated mechanically. This was followed by centrifugation at 2000 rpm for 3 min to precipitate the aggregates, resulting in a homogeneous colloidal dispersion. LCs used were either pure 4‐cyano‐4′‐pentylbiphenyl (5CB, from EM Chemicals) for the nematic phase or 5CB with a chiral additive, cholesterol pelargonate (from Sigma Aldrich), for the cholesteric phase. The concentration of the chiral additive was varied to obtain the cholesteric pitch in the range of 20–50 µm. LC cells were constructed by infiltrating the colloidal dispersions in between two polyimide‐coated indium tin oxide (ITO) glass slides with the ITO side facing inwards to allow for the application of electric field. Silica spacers in UV‐curable glues placed between the glass slides controlled the cell thickness (10–50 µm) and held the slides together after UV irradiation; the polyimide coated slides were rubbed unidirectionally to define planar boundary conditions and orientation of **N**_0_.

In addition, cellulose nanocrystals (CNCs) were also used to align GNPBs in order to measure the extinction spectra with polarization dependence, by following the approach and CNC preparation procedures described in Ref. [26] for gold nanorods. The mixture containing PEG‐capped GNPBs and 3 wt% CNC solution was first sonicated for 30 min and then applied dropwise on a glass slide pretreated with Piranha solution (H_2_SO_4_:H_2_O_2_ = 3:1, volume ratio). The drops were spread and sheared by moving another glass slide over close to the glass substrate. The process was repeated until ≈200 µL of the mixture was used and a layer of coaligned GNPBs and CNCs formed on the substrate.^[^ ^26^ ^]^

### Experimental Characterization

TEM images and energy dispersive spectrometer (EDS) elemental distribution maps were acquired with an FEI Talos F200X. The images of LC cells with the GNPBs dispersion in LCs were captured using a polarizing optical microscope (Olympus BX‐51) equipped with 10×, 20×, 50× air objectives (Olympus) and a charge‐coupled device (CCD) camera (PointGrey Grasshopper3). Dark‐field optical microscopy imaging was performed utilizing an oil‐immersion dark‐field condenser (NA = 1.2). The polarization dependence of extinction spectra was probed by a spectrometer (Ocean Optics, USB2000‐FLG) mounted on the microscope with a rotatable polarizer to define the angle between the light polarization and **N**_0_. To monitor the switching of the LC‐GNPB composite when the electric field is applied, transmitted light in the vicinity of the long‐wavelength SPR peak was selected by a bandpass filter (780/20 nm, Semrock Inc.) and measured with a photodiode (Thorlabs, PDA100A). Homemade LabVIEW programs controlled a data acquisition board (National Instrument, SCC‐68) to generate the electric field and acquire the signal from the photodiode. Heliknotons were generated and manipulated by holographic laser tweezers which is based on an ytterbium‐doped fiber laser (YLR‐10‐1064, IPG Photonics, operating at 1064 nm) and a phase‐only spatial light modulator (P512‐1064, Boulder Nonlinear Systems) integrated with an Olympus inverted optical microscope IX81.

### Numerical Modeling and Visualization of Topological Structures

Topological structures in chiral LC‐GNPB were modeled by numerically minimizing the Frank–Oseen free energy functional^[^ ^1^, ^25^, ^38^ ^]^

(1) $$\begin{array}{ccc} F & = & F_{\mathrm{elastic}}+F_{\mathrm{electric}}\\ & = & \int d^{3}r\left\{\frac{K_{11}}{2}{\left(\nabla \cdot N\right)}^{2}+\frac{K_{22}}{2}{\left[N\cdot (\nabla \times N)\right]}^{2}+\frac{K_{33}}{2}{\left[N\times (\nabla \times N)\right]}^{2}\right.\\ & & \left.+\;\frac{2\pi K_{22}}{p}N\cdot \left(\nabla \times N\right)\right\}-\frac{\varepsilon_{0}\Delta \varepsilon }{2}\int d^{3}r{\left(E\cdot N\right)}^{2} \end{array}$$

where *K*_11_ =  6.4 pN, *K*_22_ =  3 pN, *K*_33_ =  10 pN are the splay, twist and bend elastic constants of 5CB (average elastic constant *K*  =  6.47 pN),^[^ ^38^ ^]^ and *p* is the equilibrium cholesteric pitch. The saddle‐splay deformation and surface energy are not included by assuming strong boundary conditions on the surfaces, consistent with experiments. The electric term describes the coupling between LC director **N**(**r**) and the applied electric field, where $\varepsilon_{0}$ is the vacuum permittivity, Δε=13.8 is the LC dielectric anisotropy and **E** is the electric field. Equilibrium heliknoton structures were obtained by a variational‐method‐based energy minimization routine previously described.^[^ ^25^ ^]^ Briefly, **N**(**r**) is updated iteratively from an initial structure using the Euler–Lagrange equation derived from Equation (1). The relaxation is terminated when the spatial average of functional derivatives decreases, over iterations, to a threshold value for the steady‐state stopping condition, indicating an energy minimum is attained. The computational volume is 4*p* ×  4*p* ×  2*p* and sampled isotropically by a cubic grid at 24 grid points per cholesteric pitch. Periodic boundary conditions and unidirectional planar boundary conditions were imposed in the lateral directions and at the top/bottom, respectively. The nonpolar helical axis field ***χ*** (**r**) which **N**(**r**) twists around was derived from the as relaxed heliknoton structure by identifying the twist axis at all spatial coordinates with the eigenvector of the local chirality tensor *C*_ij_ = *N*_k_ *ε*_ljk_∂_i_ *N*_l_.^[^ ^25^ ^]^ The singular vortex line in the immaterial field was determined by finding connected spatial regions where ***χ*** (**r**) is ill‐defined.

### Computer Simulations of Surface Plasmon Resonance Effects

The finite‐difference time‐domain (FDTD) method was used to computer‐simulate the field enhancement of the gold bipyramid (Figures 1c and 3e), which was modeled as two connecting pentahedrons with their bottom surface joined together and having rounded tips on both ends along the C_5_ axis. The gold bipyramid was taken to have the following geometric parameters: the effective radius at the equator *R* = 17.5 nm, the total length along the C_5_ axis *h* = 70 nm and the radius at the tips *r* = 3.5 nm, consistent with the TEM images. The refractive indices of the 5CB host^[^ ^12^ ^]^ were used to calculate the background index of the nematic host to describe the optical‐frequency dielectric properties of the host LC environment. A plane wave was incident towards the bipyramid for two orthogonal directions, i.e., along the C_5_ axis of the bipyramid and perpendicular to it. In each case, different linear polarizations of the incident light were also considered (Figure 3e–i). The grid size was taken to be 0.5 nm and the number of grid points was 7.58467 × 10^6^.

In addition, surface plasmon resonance extinction spectra corresponding to a single GNBP in 5CB were modeled using the NanoHub online platform (https://nanoHUB.org ). The geometry of GNBP (35 nm by 70 nm, consistent with the TEM image) was generated by use of the nanoDDSCAT+ tool,^[^ ^39^ ^]^ which takes a shape input in the form of a triangulated mesh and converts it into a cubic array of points describing positions of the dipoles, providing the input for DDSCAT. An interdipole spacing of 0.7 nm was used. The bulk experimental dielectric functions of Au from Johnson and Christy were utilized without any corrections.^[^ ^40^ ^]^ Extinction spectra in the 500–1000 nm wavelength range were obtained from each simulation at different wavelengths. The GNBP was excited with a plane wave propagating along the thickness of the GNBP and linearly polarized at different angles relative to the long axis of the particle. The effective refractive index of the LC medium was calculated as that of a uniaxial optical crystal with extraordinary and ordinary refractive indices *n*_e_ = 1.74 and *n*_o_ = 1.54, respectively.

## Conflict of Interest

The authors declare no conflict of interest.

## Author Contributions

Z.M. synthesized and surface‐functionalized gold nanoparticles and performed experimental characterizations. B.L. and H.L. did the analysis of polarized surface plasmon resonance spectra. B.S. and Y.Y. contributed to electro‐optic characterization of colloidal dispersions and analyzed data. J.‐S.T. did numerical modeling and assisted with imaging experiments involving the topological solitons. Y.W., G.Z., and I.I.S. initiated, designed and supervised the study and wrote the manuscript, with feedback from all authors.

## Supporting information

Supporting Information

Supplemental Video 1

Supplemental Video 2

## Data Availability

The data used to support the findings of this study are available from the corresponding author upon request.
