# Supplementary material for: Nematic Order, Plasmonic Switching and Self‐Patterning of Colloidal Gold Bipyramids
Source: Adv Sci (Weinh). 2021 Sep 20;8(22):2102854. doi: 10.1002/advs.202102854 (PMC8596134; doi:10.1002/advs.202102854)
Supplement: Supplementary file 1 — Supporting Information [file ADVS-8-2102854-s002.pdf]

© 2021 Wiley-VCH GmbH

ADVANCED SCIENCE
Open Access

Supporting Information

for *Adv. Sci.,* DOI: 10.1002/advs.202102854

Nematic Order, Plasmonic Switching and Self-patterning of Colloidal Gold Bipyramids

Zhijian Mai, Ye Yuan, Jung-Shen B. Tai, Bohdan Senyuk, Bing Liu, Hao Li, Yao Wang, Guofu Zhou*, and Ivan I. Smalyukh**

Supporting information

The Supporting Information is available free of charge on the ACS Publications website.

Supporting figures providing additional characterization of nanoparticles

Movie S1: Polarization dependence of color appearance of the LC-GNPB colloidal dispersions. (mp4 file)

Video shows a nematic LC cell of 30 μm in thickness with GNPBs at volume fraction of about 1.0 wt%. The color of the LC cell changes during rotation because of the polarization dependence of SPR spectra of the LC-GNPB colloidal dispersions.

Movie S2: Electric switching and polarization dependence of LC colloidal dispersions (mp4 file)

This video shows switching of a nematic LC cell of 30 μm in thickness with GNPBs at volume fraction of about 1.0 wt%. The color of the LC cell changes during the switching because of the polarization dependence of SPR of LC-GNPB dispersions.
